# Branching hierarchy shapes branch–leaf allometry and trait coordination in mountain shrubs of Dongling Mountain, Beijing

**DOI:** 10.1093/aobpla/plag041

**Published:** 2026-09-22

**Authors:** He Li, Siying Zhong, Xiuchen Jiang, Jianghua Zhang

**Affiliations:** Jiangsu Normal University, Xuzhou, Jiangsu 221000, China; Jiangsu Normal University, Xuzhou, Jiangsu 221000, China; Oregon State University, Corvallis, OR 97331, United States; Xinzhou Normal University, Xinzhou, Shanxi 034000, China

**Keywords:** branch order, branch–leaf allometry, plant trait network, shrub architecture

## Abstract

Branch–leaf allometry is fundamental to understanding biomass allocation, leaf deployment, and resource-use strategies in woody plants. However, most studies have focused on trees or terminal twigs, while the role of branching hierarchy in shaping branch–leaf relationships in shrubs remains poorly understood. In this study, we investigated 12 dominant shrub species from Dongling Mountain, Beijing, China, and examined how 11 branch–leaf traits, allometric relationships, and trait coordination varied among architectural life forms, elevational zones, slope aspects, and branch orders. The results showed: except for the total leaf biomass, all other branch–leaf traits showed non-significant phylogenetic signal, and phylogenetic PCA did not reveal clear separation among life forms, elevation zones, or slope aspects, suggesting that trait variation was not primarily structured by phylogenetic relatedness. Branch–leaf traits varied significantly across ecological and architectural groups. With increasing branch order, branch cross-sectional area, branch length, branch volume, total leaf biomass, and branch biomass decreased, whereas mass-based and volume-based leafing intensity increased. Branch–leaf allometric relationships were scale-dependent: relationships that were allometric at the whole-plant level tended to become isometric or near-isometric at the branch level. Individual leaf mass was negatively associated with leafing intensity, supporting a leaf size–number trade-off in shrubs. Plant trait networks further showed that lower-order branches were dominated by simple structural covariation, whereas higher-order branches exhibited stronger coordination among branch architecture, leaf number, and biomass allocation. These findings demonstrate that branching hierarchy is a key organizational level shaping branch–leaf allometry and trait coordination in mountain shrubs. Considering branch order can therefore improve our understanding of shrub resource allocation strategies and architectural adaptation in heterogeneous mountain environments.

## Teaser

Shrubs are built from branches of many kinds—thick basal stems and slender leafy twigs—yet are often studied as if all branches were alike. Across 12 shrub species on a Beijing mountain, we found that the rules linking branch and leaf size change with a branch's position in the plant. Branch order is a key level organizing how shrubs build their crowns, and comparative studies of plant form should not overlook it.

## Introduction

The relationship between branch and leaf size is an important dimension of plant life-history strategies (Westoby and Wright 2003, 2006). This scaling relationship reflects biomass allocation between supporting and photosynthetic organs and is closely linked to variation in plant physiological ecology (Niklas and Enquist 2002, Pickup et al. 2005). Most empirical studies have reported a significant positive correlation between twig or branch size and leaf size (Sun et al. 2006, Yang et al. 2009), but whether this relationship follows an allometric or isometric pattern remains debated. For example, branch cross-sectional area (BCA) has been found to scale allometrically with total leaf area and individual leaf area in woody species from Changbai Mountain (Sun et al. 2006), whereas mechanical support constraints may lead to an isometric relationship between BCA and leaf area (Brouat and McKey 2001). Compared with trees, however, branch–leaf scaling relationships in shrubs remain less well understood (White 1983, Sun et al. 2006, Yang et al. 2009). Given the high branching density, strong sprouting capacity, and ecological importance of shrubs, especially in terrestrial carbon dynamics (Hu et al. 2006, Xie et al. 2019), investigating branch–leaf allometry in shrubs can improve our understanding of resource allocation and carbon gain strategies.

Plants must allocate limited resources through various trade-off strategies, among which the trade-off between leaf size and leaf number (LN) is particularly important. Under stressful conditions such as drought, low temperature, or high radiation, plants often produce smaller leaves, whereas larger leaves may be favoured under shaded, moist or nutrient-rich conditions to maximize light interception and carbon acquisition (Parkhurst and Loucks 1972, Givnish 1987, Westoby and Wright 2003, Wright et al. 2017). The ‘leafing-intensity premium’ hypothesis proposes that selection for higher leafing intensity—the number of leaves borne per unit of stem or branch biomass or volume—inevitably results in smaller leaf size (Kleiman and Aarssen 2007). Numerous studies have shown a negative relationship between leaf size and leafing intensity across plant species (Kleiman and Aarssen 2007, Yang et al. 2008, Milla 2009, Sun et al. 2019), although both isometric and allometric patterns may occur (Scott and Aarssen 2012). Most of these studies have focused on trees or herbaceous species. Therefore, examining whether shrub species follow similar leaf size–number trade-offs, and whether these trade-offs vary across environmental gradients such as elevation and slope aspect, is important for understanding shrub adaptation in mountain environments.

Shrubs differ from trees and herbs in whole-plant architecture and branching habits. They commonly possess multiple woody stems arising near the ground, and their growth forms may vary from erect and discrete to spreading, prostrate, tangled or densely clumped forms. Such architectural variation may influence how branch units are organized, how leaves are displayed, and how biomass is allocated between supporting and photosynthetic organs. Plant architecture provides a multilevel framework for understanding plant form, branching pattern and growth habit (Barthélémy and Caraglio 2007, Chomicki et al. 2017), and architectural traits are increasingly recognized as important components of trait-based ecology because they link plant form to functional responses to environmental conditions (Laurans et al. 2024). Accordingly, in this study, shrub species were classified into three architectural life forms based on branch discreteness and crown organization: distinct and discrete branching, tangled and spreading branching, and dense clustered branching. This classification allowed us to test whether shrub architectural differences are associated with variation in branch–leaf allometry and trait coordination.

Beyond whole-plant architectural differences, branching hierarchy itself may further shape branch–leaf relationships. Different branch orders often differ in their structural, hydraulic, and leaf-supporting functions. Terminal branches primarily support leaves and contribute directly to photosynthetic display (Coble and Cavaleri 2015), whereas basal or lower-order branches play greater roles in mechanical support, hydraulic transport, and construction of the crown’s spatial framework (i.e. the positioning of higher-order branches and leaves in space). Nevertheless, most studies on branch–leaf size relationships and leaf size–number trade-offs have focused primarily on terminal twigs or current-year shoots (Westoby and Wright 2003, Sun et al. 2006), with limited attention to how these relationships change across branching hierarchies. This limitation is particularly important for shrubs, where the definition of a scalable branch unit can be more complex than in trees. If different branch orders differ in their functional roles, branch–leaf allometry and leaf size–number trade-offs may not be consistent from the whole-plant level to different branch levels.

In addition to pairwise allometric relationships, shrub branch–leaf strategies should be evaluated in both phylogenetic and multivariate contexts. Closely related species may share similar branch construction, leaf deployment or biomass allocation traits due to common ancestry, and phylogenetic comparative methods can help distinguish ecological or architectural differentiation from phylogenetic constraint (Harvey and Pagel 1991, Cornwallis and Griffin 2024). Moreover, pairwise scaling analyses cannot fully reveal how multiple branch and leaf traits are coordinated simultaneously. Plant trait networks provide a complementary framework for identifying trait coordination, trade-offs and hub traits in multivariate trait space (He et al. 2020, Wang et al. 2023). Therefore, combining allometric analysis, phylogenetic signal tests, phylogenetic PCA and plant trait network analysis can improve our understanding of how branching hierarchy shapes branch–leaf allometry and trait coordination.

In this study, we investigated 12 dominant shrub species from Dongling Mountain, Beijing, and classified them by architectural life form, elevational zone, slope aspect and branch order. We first assessed whether branch–leaf traits were strongly influenced by phylogenetic relatedness, and then examined branch–leaf allometry and trait coordination across different organizational and environmental contexts. Specifically, we addressed three questions: (1) How do branch–leaf traits and allometric relationships vary among shrub life forms, environmental gradients (elevation and slope aspect) and branch orders? (2) Are leaf size–number trade-offs consistent between the whole-plant level and different branch orders? (3) How does branching hierarchy affect the coordination among branch architecture, leaf deployment and biomass allocation? We expected that lower-order branches would be more strongly associated with structural and hydraulic constraints, whereas higher-order branches would show stronger integration between leaf deployment and biomass allocation. Overall, this study aims to clarify how branching hierarchy shapes branch–leaf allometry and trait coordination in mountain shrubs.

## Materials and methods

### Study area

**Figure 1:**
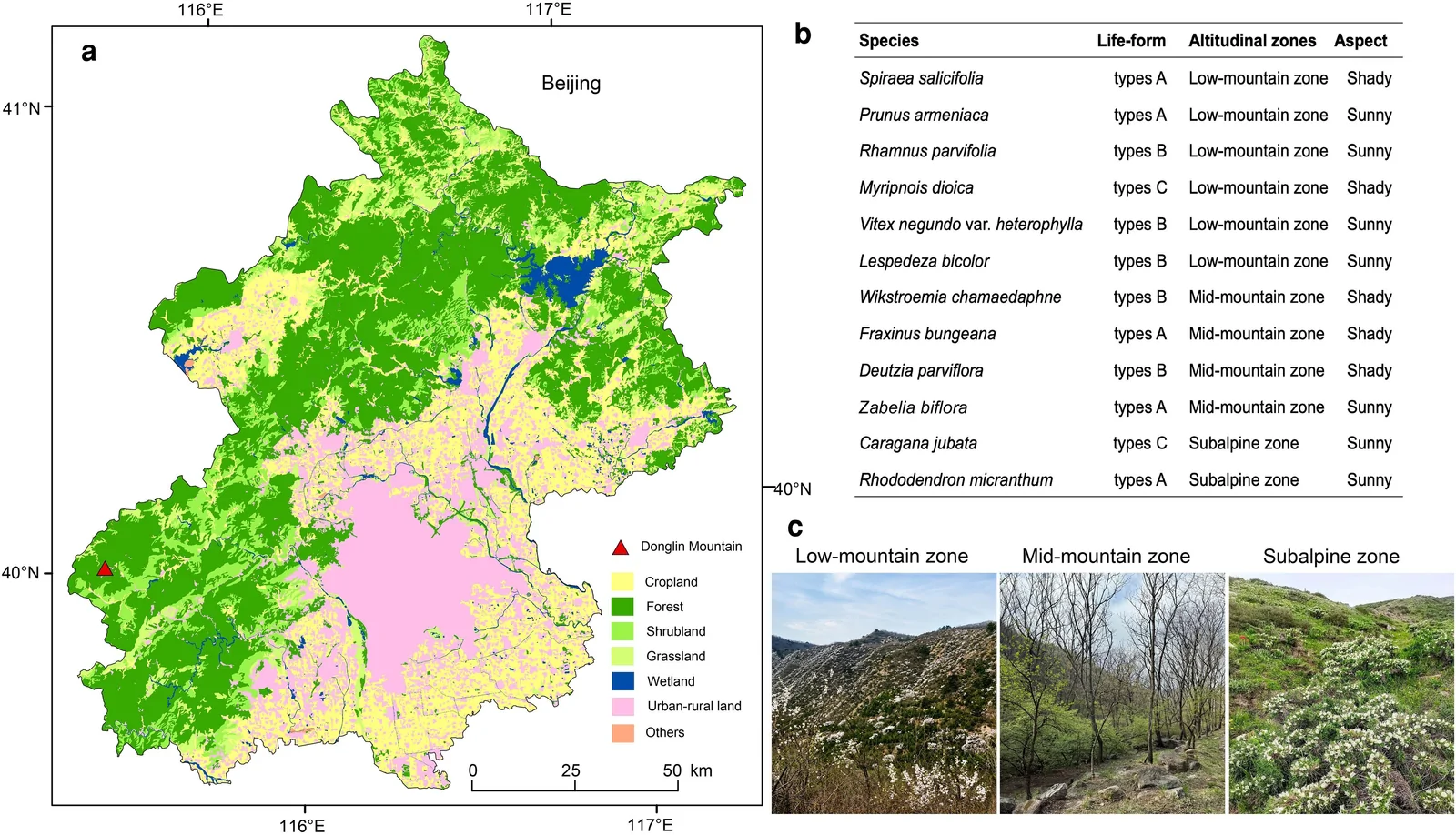
(a) Location map of the study area, (b) basic information of the 12 shrub species included in this study, and (c) pictures of altitude zones.

This study was conducted in Dongling Mountain, Beijing, China, which is located at 39°48′–40°00′N and 115°24′–115°36′E. The study area covers approximately 60 km^2^ and reaches a maximum elevation of 2303 m a.s.l. (Fig. 1). The region has a temperate continental monsoon climate, with a mean annual temperature ranging from 2°C to 7°C and mean annual precipitation of 400–500 mm. The dominant soil types include mountain brown soil, subalpine meadow soil and cinnamon soil. Vegetation is primarily composed of temperate deciduous broad-leaved forests and shows a pronounced vertical distribution pattern. Shrublands are mainly distributed in low-elevation areas below 900 m and are dominated by species, such as *Vitex negundo* var. *heterophylla*, *Spiraea salicifolia*, and *Prunus armeniaca* (Lou 2004).

### Sample collection and trait measurements

**Figure 2:**
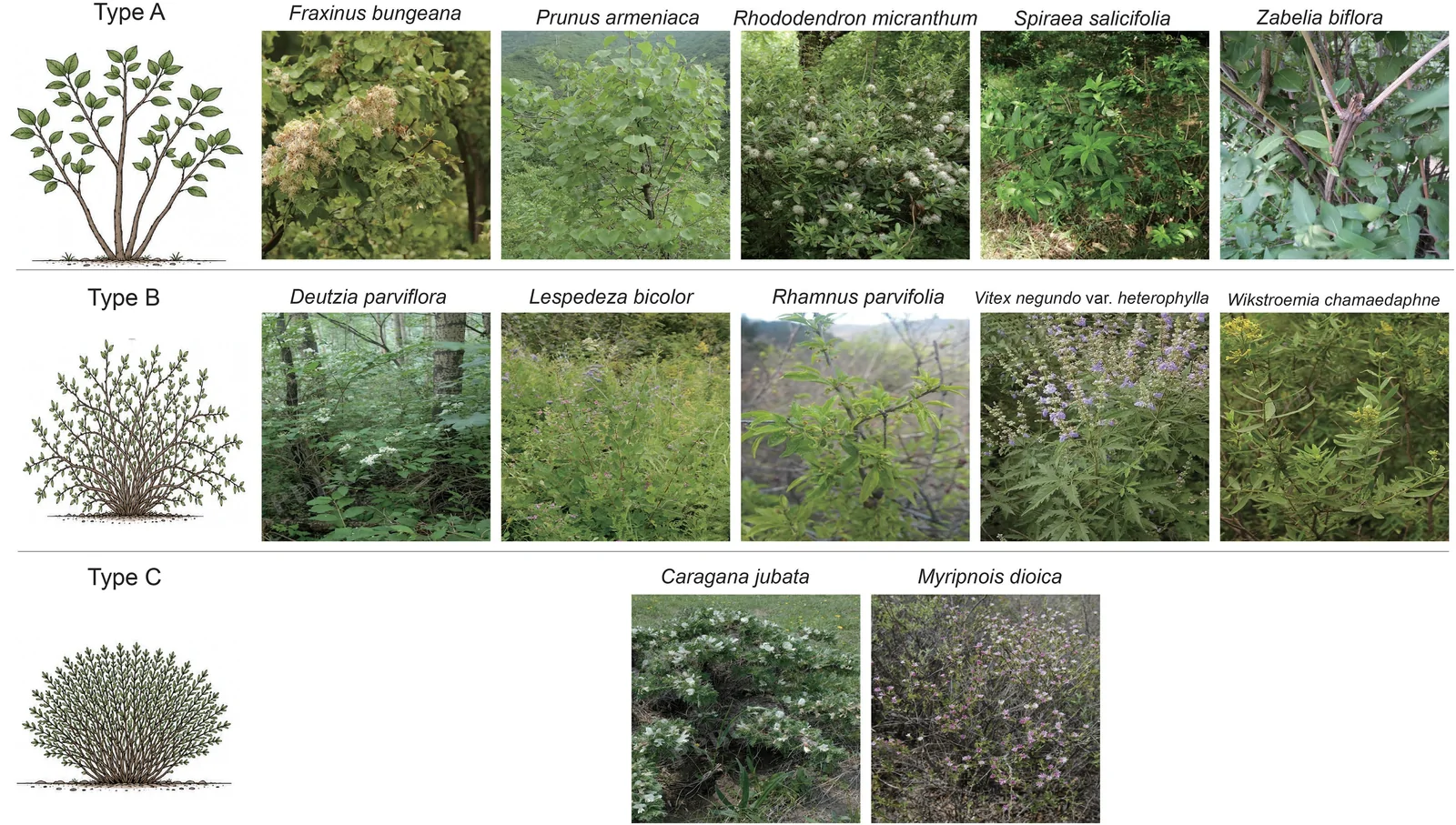
Schematic diagram of the three architectural life forms and photographs of 12 dominant shrub species sampled at Dongling Mountain, Beijing. Type A, distinct and discrete branching; Type B, tangled and spreading branching; Type C, dense clustered branching, classified after Xie et al. (2019).

Field sampling was conducted in June 2013 on shrub-dominated slopes in Dongling Mountain, where species composition and habitat conditions were relatively homogeneous. For each shrub species, 6–7 individuals were selected, and species identity and plant height were recorded. In total, 74 shrub individuals from 12 species were sampled (Fig. 2).

Branches were classified hierarchically using a centrifugal ordering method. The main shoot or whole shoot system was designated as the 0th-order branch. Branches directly sprouting from it were classified as first-order branches; branches emerging from first-order branches were defined as second-order branches, and so forth, until terminal branches without further subdivisions were reached. Starting from the terminal branches, all branches were sequentially clipped according to branch order. For small shrubs, all leaves were collected. For larger branches, a subset of leaves was randomly sampled and sealed in plastic bags, and the total number of leaves was recorded. In addition, three branches were randomly selected from each individual shrub, and 5- to 8-cm stem segments were clipped from the middle portion of each branch for stem density (SD) measurements. All branch and leaf samples were transported to the laboratory for trait measurements.

Leaf traits included LN, total leaf biomass (TLB, g), individual leaf mass (ILM, g), while branch traits included branch diameter (BD, cm), branch length (BL, cm), and bark thickness (mm). BCA (cm^2^) and branch volume (BV, cm^3^) were calculated by approximating branches as cylindrical structures with circular cross-sections. Stem volume was measured using the water displacement method. After volume measurement, stem segments were oven-dried at 75°C to constant mass and weighed using a precision electronic balance (Sartorius AG, Goettingen, Germany; precision: 0.0001 g). SD (g cm^−3^) was calculated as the ratio of dry mass to stem volume. Trait measurements followed standard protocols for plant functional traits where applicable (Pérez-Harguindeguy et al. 2013).

All leaf and branch samples were oven-dried at 75°C to constant mass and weighed to determine leaf biomass and branch biomass for each branch order. Based on the recorded LN, ILM (g) was calculated as an index of leaf size. Two indices of leafing intensity were also calculated: mass-based leafing intensity (MLI, leaves g^−1^), defined as LN per unit branch biomass, and volume-based leafing intensity (VLI, leaves cm^−3^), defined as LN per unit BV (Kleiman and Aarssen 2007, Sun et al. 2019).

### Statistical analyses

Based on branching morphology, the 12 shrub species were classified into three architectural life-form types following Xie et al. (2019): Type A, shrubs with distinct, countable and discrete branches; Type B, shrubs with indistinct, tangled and spreading branches; and Type C, shrubs with indistinct branches forming dense clusters. Shrub species were also classified into three elevational zones following Lou (2004): low-mountain zone (<900 m), mid-mountain zone (900–2000 m), and subalpine zone (>2000 m). Based on slope aspect, shrubs were further classified as sunny-slope or shady-slope species (Figs 1 and 2). For individual-level analyses, branch and leaf traits were averaged across all branches within each individual shrub. For branch-order-level analyses, traits were calculated separately for each branch order. All continuous traits were log_10_-transformed before statistical analyses to improve normality and reduce the influence of extreme values.

To evaluate whether branch–leaf traits were influenced by phylogenetic relatedness, phylogenetic comparative analyses were conducted at the species level. Species-level mean trait values were calculated and log_10_-transformed before analysis. The phylogenetic tree for the 12 species was generated by pruning the dated seed-plant megaphylogeny GBOTB (Smith and Brown 2018) to the study species using the V.PhyloMaker2 package (Jin and Qian 2022) in R, with species bound to their genus- and family-level nodes and BLs retained from the megaphylogeny. Phylogenetic signal was quantified using Pagel’s *λ* and Blomberg’s *K*, and their significance was assessed using likelihood-ratio and randomization tests, respectively (Pagel 1999, Blomberg et al. 2003). Phylogenetic principal component analysis was performed using the log_10_-transformed species-level trait matrix and the phylogenetic tree to visualize species distribution in phylogenetically corrected multivariate trait space. Species were coded by life form, elevational zone, and slope aspect to assess whether ecological groups were separated after accounting for phylogenetic relatedness. Phylogenetic analyses were conducted using the ape and phytools packages in R (Paradis et al. 2004, Revell 2012, 2024).

One-way analysis of variance (ANOVA), followed by least significant difference (LSD) multiple comparisons, was used to test for differences in branch and leaf traits among shrub life forms, elevational zones, slope aspects and branch orders. The relationships between branch and leaf traits were described using the allometric equation:


$$Y=\beta X^{\alpha }$$

where *X* and *Y* are paired branch and leaf traits, *α* is the scaling exponent, and *β* is the normalization constant. After log10 transformation, the equation was expressed as: $\log_{10}\;(Y)=\log_{10}\;(\beta )+\alpha \log_{10}\;(X)$. A scaling exponent |α|=1 indicates an isometric relationship, whereas a significant deviation from 1 indicates an allometric relationship (Niklas 1994, Warton et al. 2006). Standardized major axis regression was performed using the smatr package in R to estimate scaling exponents for the relationships between leaf biomass and branch biomass, ILM and leafing intensity, BCA and ILM, and BCA and leafing intensity. Both MLI and VLI were used. Deviations of scaling exponents from |α|=1 and differences in scaling relationships among shrub life forms, elevational zones, slope aspects, and branch orders were tested using the procedures implemented in smatr (Warton et al. 2012). To assess whether the branch- and individual-level allometric relationships were sensitive to non-independence among individuals of the same species, we repeated the standardized major axis analyses on species mean trait values (geometric means of log_10_-transformed traits; *n* = 12 species), so that each species contributed a single point (Supplementary Table S5).

To further evaluate multidimensional coordination among branch and leaf traits, plant trait networks were constructed using the MultiTraits package in R (He et al. 2026). Eleven branch–leaf traits were included: BD, BL, BV, BCA, ILM, SD, MLI, VLI, TLB, branch biomass, and LN. All traits were log_10_-transformed and standardized before network construction. Following the plant trait network framework, traits were represented as nodes and significant trait associations were represented as edges (He et al. 2020). Pairwise Pearson correlations were calculated among traits, and significant associations with |*r*| > 0.30 and *P* < 0.05 were retained as network edges. Positive correlations were interpreted as trait coordination, whereas negative correlations were interpreted as potential trade-offs. Network-level metrics, including number of edges, network density, average clustering coefficient, average path length, and modularity, were calculated, and hub traits were identified based on node degree. Network metrics and graph objects were processed using MultiTraits and igraph where necessary (Csárdi and Nepusz 2006).

All statistical analyses were conducted in R version 4.3.2 (R Core Team 2023).

## Results

### Phylogenetic structure and trait independence

**Figure 3:**
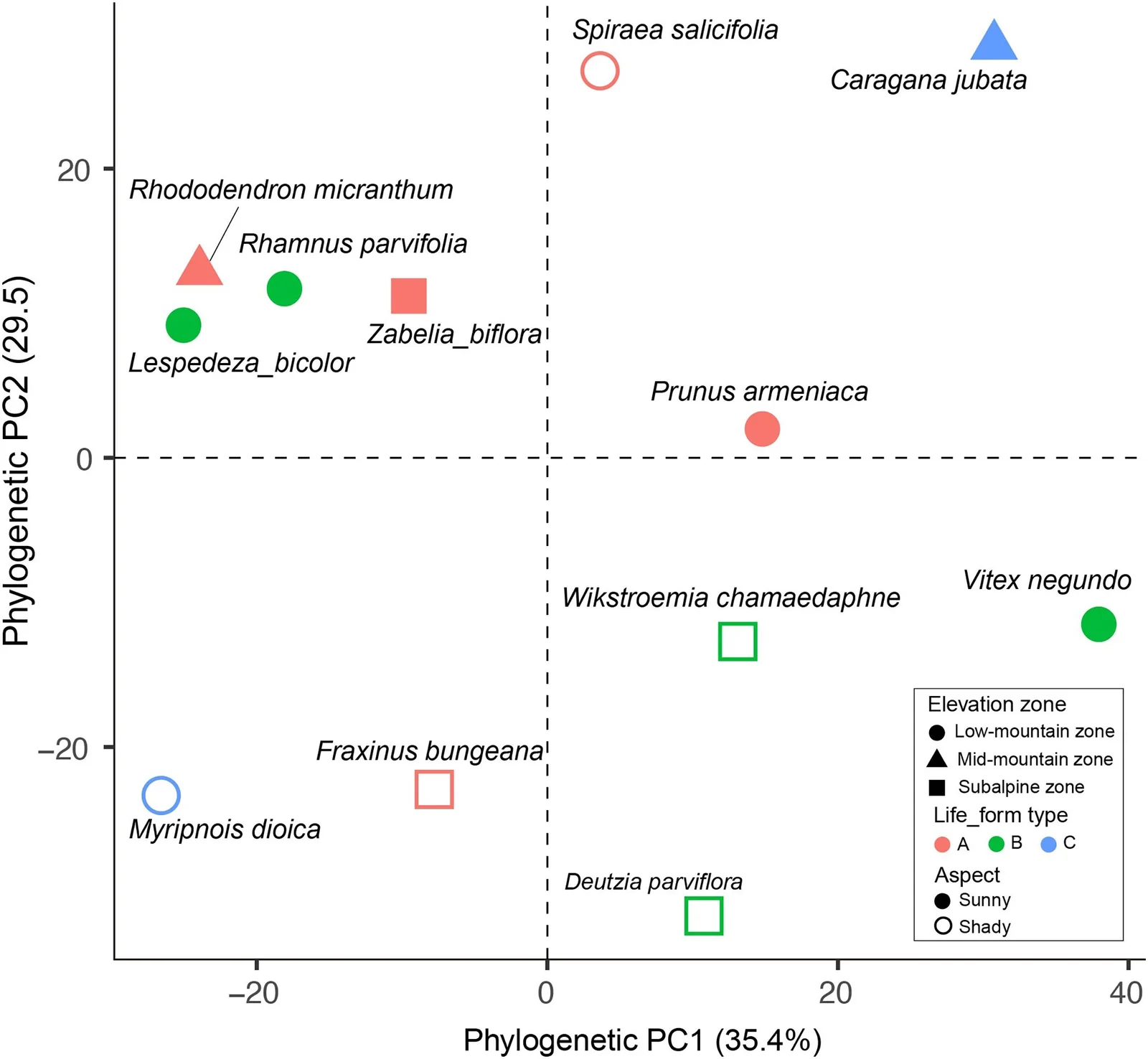
Phylogenetically corrected principal component analysis (pPCA) of branch–leaf traits across 12 shrub species. Species are coded by architectural life-form type (a–c), elevation zone (low, mid, subalpine), and slope aspect (sunny, shady).

Most branch–leaf traits showed weak or non-significant phylogenetic signal (Supplementary Table S1). Pagel's *λ* was close to zero and non-significant for most traits, and Blomberg's *K* values were generally at or below 1. TLB was the only trait with a significant signal under Blomberg's *K* (*K* = 1.30, *P* = 0.009), although its Pagel's *λ* was not significant (*λ* = 1.09, *P* = 0.059); VLI showed a relatively high but non-significant Blomberg's *K* (*K* = 1.07, *P* = 0.098). Consistent with these tests, the phylogenetic PCA showed no clear separation among life forms, elevation zones or slope aspects (Fig. 3): species from different architectural and environmental groups largely overlapped in the phylogenetically corrected trait space, and although some species occupied distinct positions along the first two phylogenetic principal components, these did not correspond to group-level separation.

### Variation in branch–leaf traits across life forms, environmental gradients and branch orders

Branch–leaf traits varied significantly among architectural life forms, elevational zones, slope aspects, and branch orders, although the affected traits differed among factors (Table 1, Supplementary Table S2). Architectural life form significantly affected all examined traits, with Type C shrubs showing greater TLB and MLI but lower ILM than Type A and Type B shrubs. Elevational zone significantly affected BD, BCA, BL, LN, and ILM, with subalpine shrubs having larger BD and BCA but shorter branches and lower ILM than those at lower elevations. Slope aspect significantly affected BD, BCA, LN, ILM, MLI, and VLI, with sunny-slope shrubs having higher ILM but lower MLI and VLI than shady-slope shrubs.

**Table 1:** *F* statistics from one-way ANOVAs testing the effects of architectural life form, elevation zone, slope aspect and branch order on 11 branch–leaf traits.

Trait	Life form	Elevation zone	Slope aspect	Branch order
*BD*	11.23*	17.50*	4.24*	34.24*
*BCA*	11.23*	17.50*	4.24*	34.24*
*BL*	3.29*	6.63*	3.02^ns^	17.35*
*BV*	6.13*	2.05^ns^	0.28^ns^	39.49*
*SD*	8.90*	3.00^ns^	2.64^ns^	0.84^ns^
*TLB*	20.90*	0.43^ns^	0.01^ns^	3.68*
*BB*	3.15*	1.69^ns^	1.32^ns^	46.82*
*LN*	96.62*	8.76*	8.58*	2.13^ns^
*ILM*	22.08*	11.01*	26.95*	1.27^ns^
*MLI*	5.48*	0.05^ns^	24.28*	16.04*
*VLI*	4.74*	0.47^ns^	16.57*	16.50*

Numerator degrees of freedom: life form, 2; elevation zone, 2; slope aspect, 1; branch order, 6. Error degrees of freedom were 284–289 for branch structural traits (BD, BCA, BL, BV, BB, SD), ≈246–251 for TLB, and ≈214–219 for LN, ILM, MLI and VLI, because not every branch bore leaves. BCA was derived directly from BD; therefore, the two traits are perfectly linearly related after log transformation and yield identical ANOVA F statistics. ns, not significant (*P* ≥ 0.05).

Trait abbreviations: BB, branch biomass; BCA, branch cross-sectional area; BD, branch diameter; BL, branch length; BV, branch volume; ILM, individual leaf mass; LN, leaf number; MLI, mass-based leafing intensity; SD, stem density; TLB, total leaf biomass; VLI, volume-based leafing intensity. Full trait values and LSD multiple comparisons are provided in Supplementary Tables S2 and S3.

^*^A significant effect (*P* < 0.05).

Branch order affected all traits except SD, LN, and ILM (Table 1, Supplementary Table S3). With increasing branch order, branch size and biomass traits (BD, BCA, BL, BV, TLB, and branch biomass) decreased, whereas MLI and VLI increased.

### Relationships between BCA, leaf biomass, and leafing intensity

**Figure 4:**
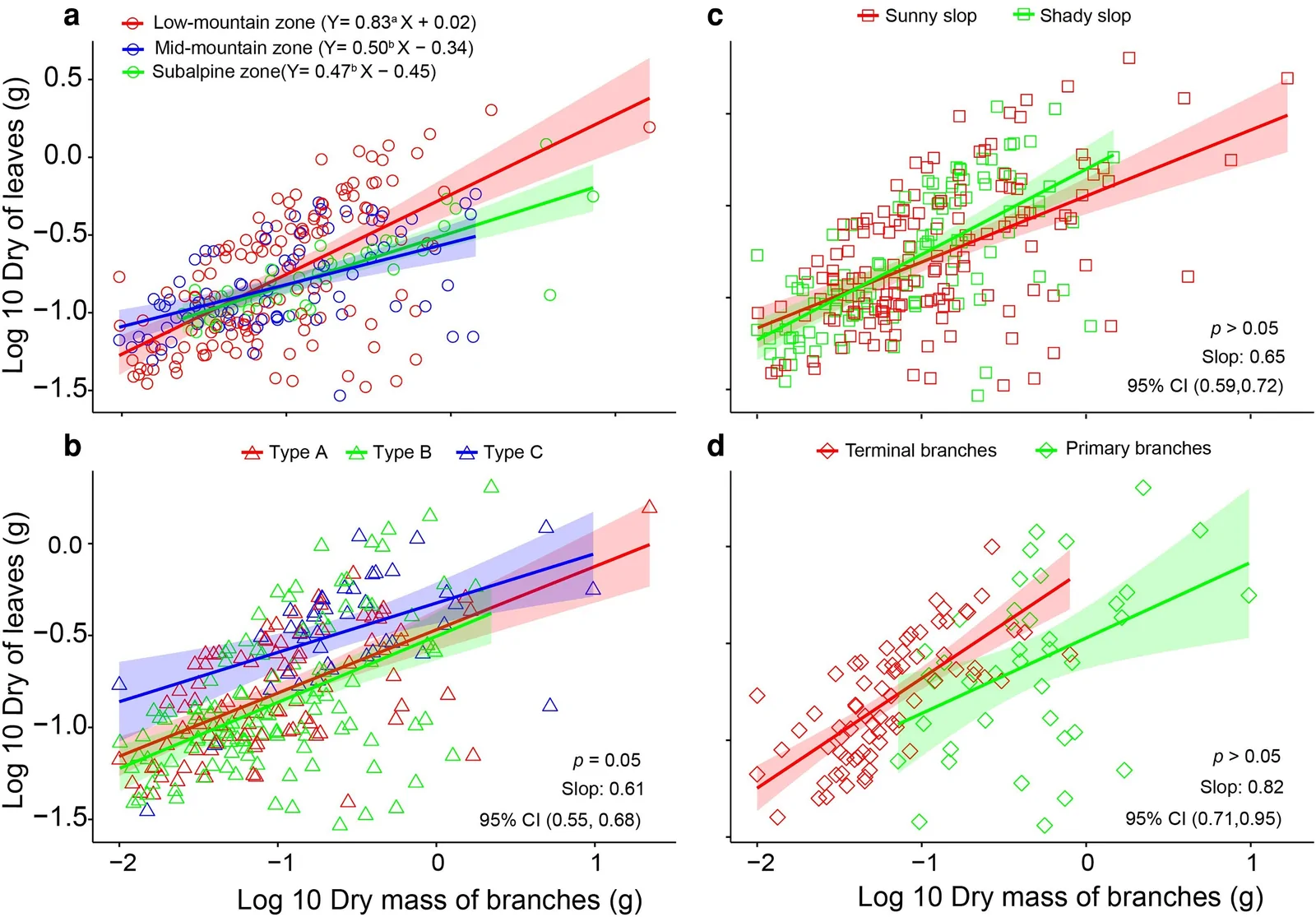
Standardized major axis (SMA) relationships between total leaf biomass and branch biomass, shown (a–c) at the individual (whole-plant) level across (a) elevation zones, (b) architectural life forms and (c) slope aspects, and (d) at the branch level for first-order versus terminal branches. All four panels share identical *x*- and *y*-axes.

**Figure 5:**
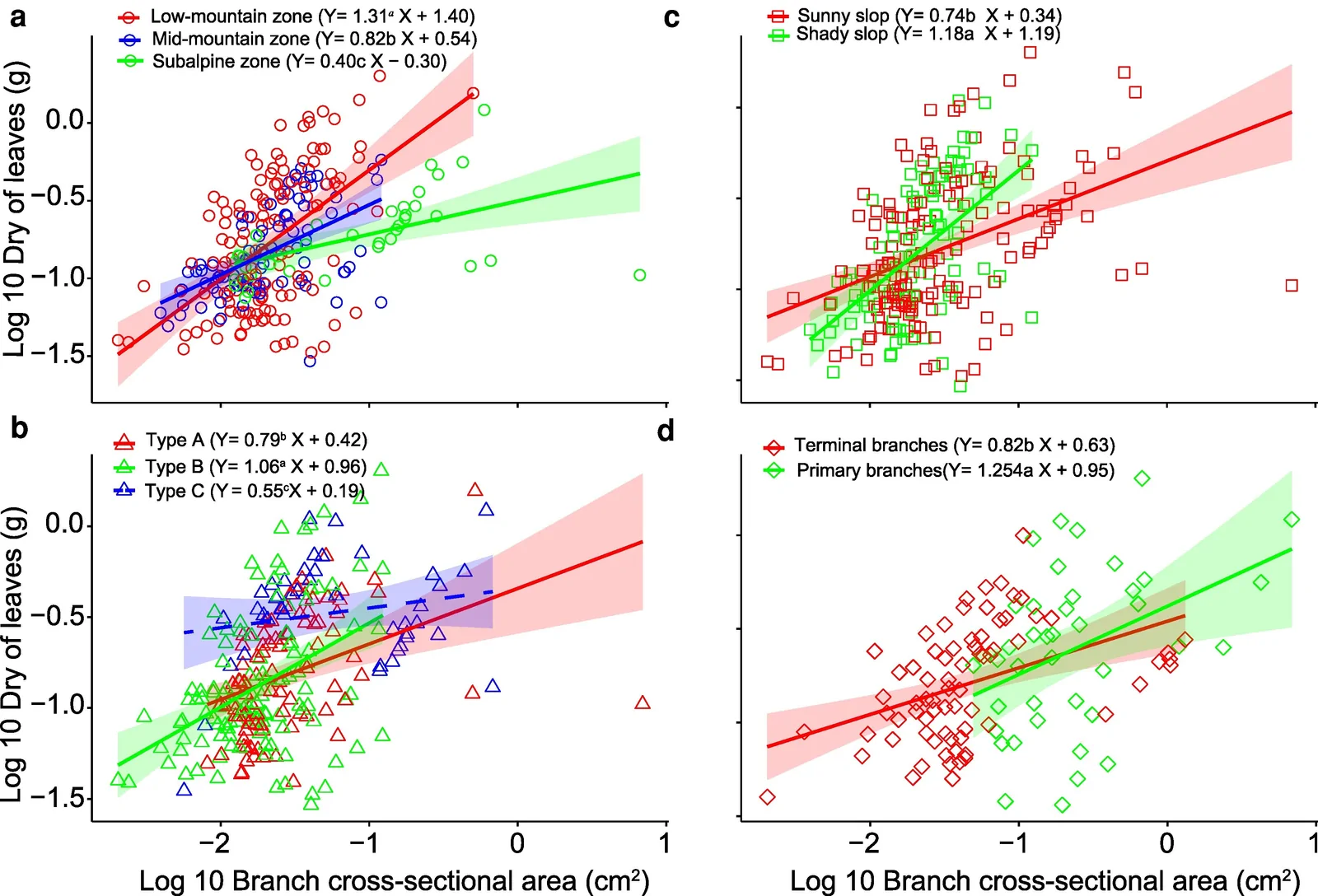
Standardized major axis (SMA) relationships between total leaf biomass and branch cross-sectional area across (a) elevation zones, (b) architectural life forms, (c) slope aspects, and (d) first-order versus terminal branches. Note: each group’s fitted SMA equation is shown, with superscript letters denoting groups whose slopes differed significantly; solid lines indicate significant regressions (*P* < 0.05) and dashed lines non-significant regressions. Slopes were additionally tested against 1.0, so that a slope indistinguishable from 1.0 indicates isometry and a slope differing significantly from 1.0 indicates allometry. The slope did not differ from 1.0 for Type B shrubs (b, *P* = 0.36) and for first-order (*P* = 0.46) and terminal (*P* = 0.06) branches (d); all other slopes differed significantly from 1.0, indicating allometric scaling.

**Figure 6:**
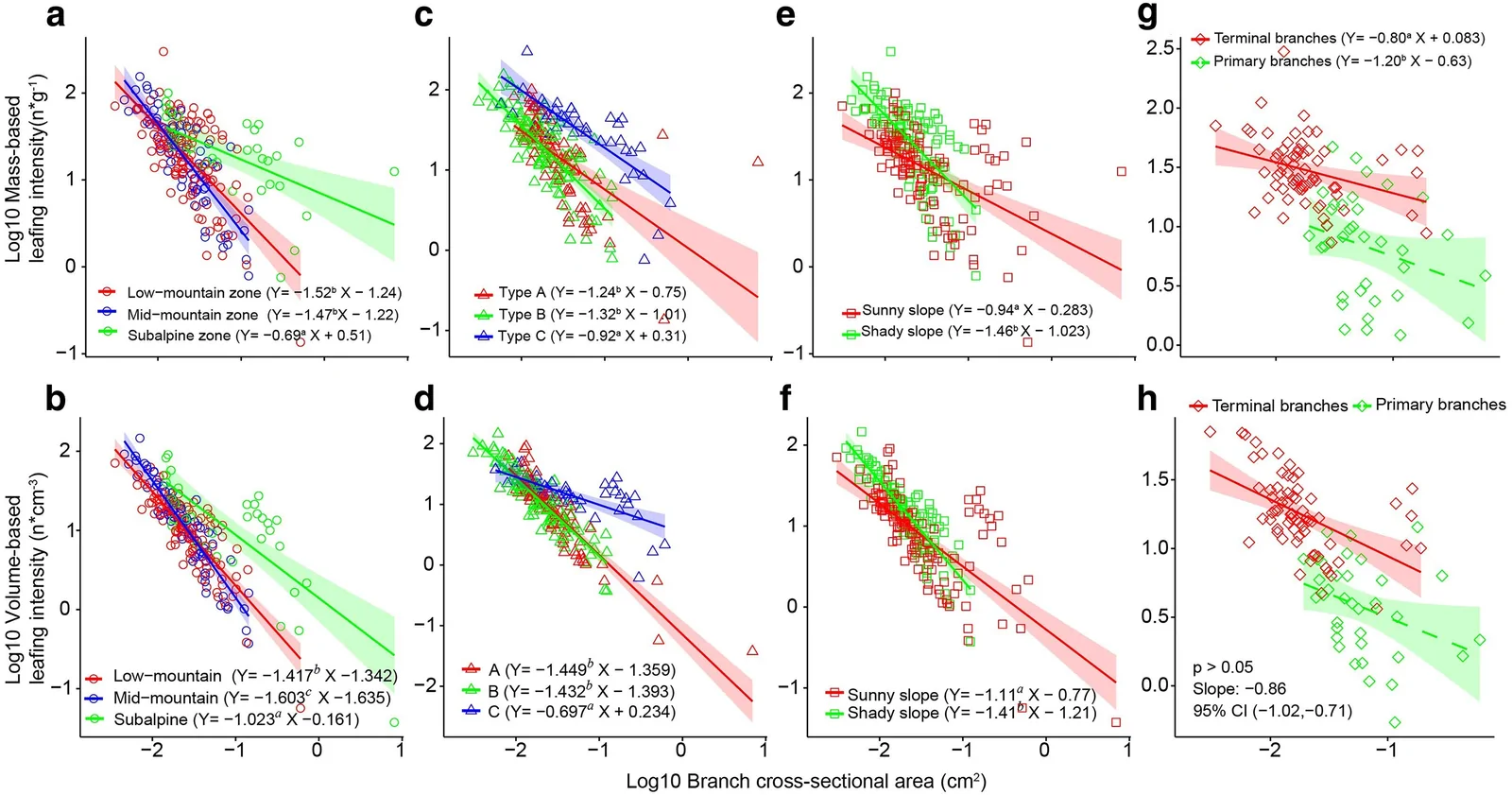
Standardized major axis (SMA) relationships between leafing intensity and branch cross-sectional area. Images (a)–(d) show mass-based leafing intensity and images (e)–(h) volume-based leafing intensity, each across (a and e) elevation zones, (b and f) architectural life forms, (c and g) slope aspects, and (d and h) first-order versus terminal branches. Note: SMA slopes were first tested for homogeneity among groups. Where slopes did not differ significantly, a single slope common to all groups is reported with its 95% confidence interval and the accompanying *P* value is the test of slope homogeneity (h). Where slopes differed, each group’s fitted equation is shown and superscript letters denote groups whose slopes differed significantly. Solid lines indicate significant regressions (*P* < 0.05) and dashed lines non-significant regressions.

**Figure 7:**
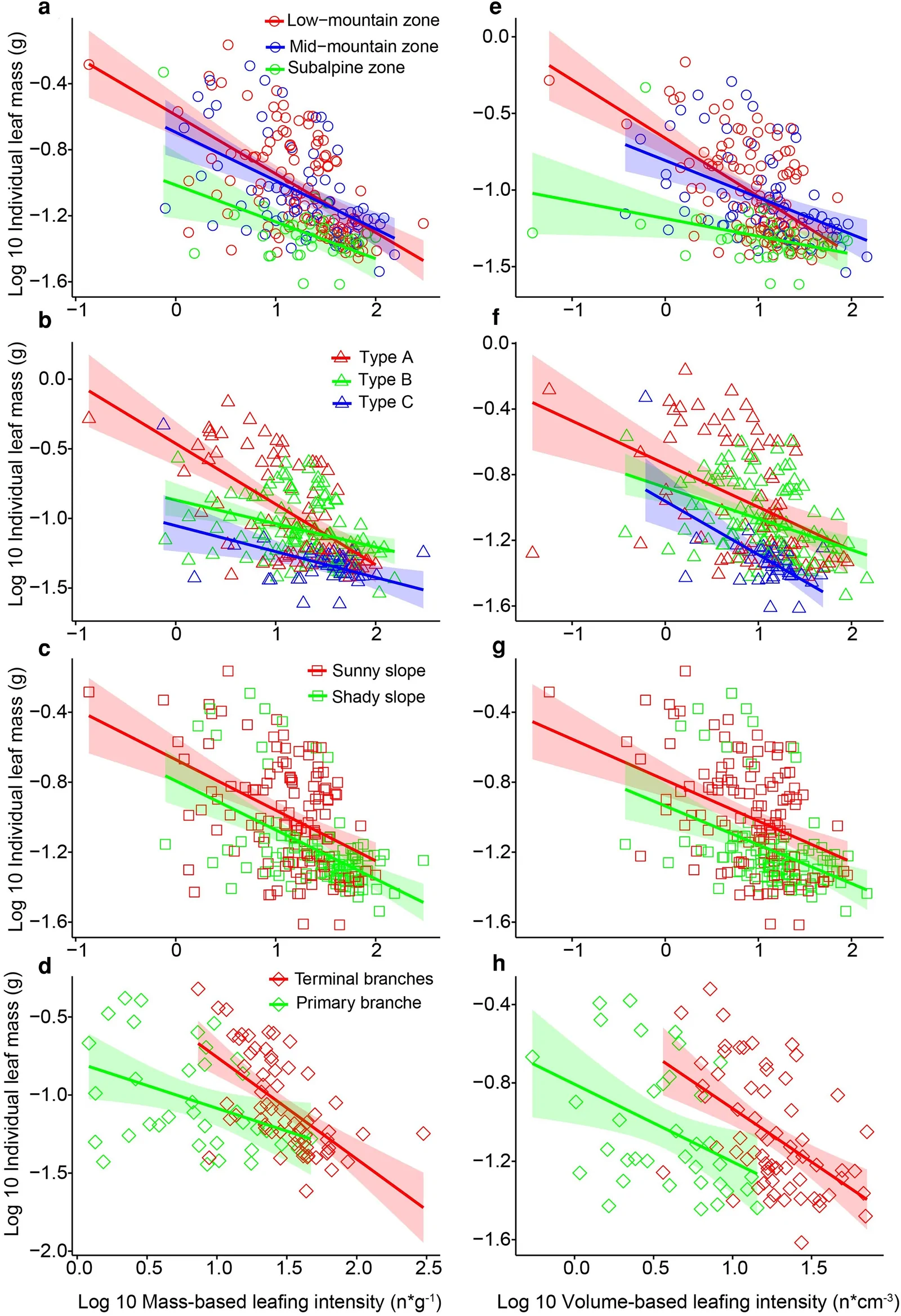
Standardized major axis (SMA) relationships between individual leaf mass and leafing intensity. Images (a)–(d) use mass-based leafing intensity and images (e)–(h) use volume-based leafing intensity, across (a and e) elevation zones, (b and f) architectural life forms, (c and g) slope aspects, and (d and h) first-order versus terminal branches. All fitted relationships are significant (*P* < 0.05), and the consistently negative slopes indicate a leaf size–number trade-off. Where group slopes differed significantly, this is indicated by superscript letters; solid and dashed lines indicate significant and non-significant regressions, respectively.

Standardized major axis regression showed that branch–leaf allometric relationships varied among organizational levels, life forms, elevational zones, slope aspects, and branch orders (Figs 4–7, Supplementary Table S4). At the individual level, TLB increased with branch biomass across elevational zones, life forms and slope aspects, with scaling slopes differing among some groups; at the branch level this relationship remained significant but was closer to isometry (Fig. 4, Supplementary Table S4). BCA was positively associated with TLB across all groups and branch orders (Fig. 5, Supplementary Table S4), with some slopes not differing from 1.0 (isometric) and others deviating significantly from isometry (allometric).

MLI and VLI were negatively related to BCA (Fig. 6, Supplementary Table S4), with slopes varying among elevational zones, life forms, slope aspects, and branch orders. ILM was negatively associated with both MLI and VLI across all groups and branch orders (Fig. 7, Supplementary Table S4).

Repeating the analyses on species means (*n* = 12) yielded the same qualitative conclusions for the leaf size–number trade-off, which remained strongly negative and significant at the species level (ILM vs MLI, slope = −1.10, *r*^2^ = 0.71, *P* < 0.05; vs VLI, slope = −1.22, *r*^2^ = 0.69, *P* < 0.05; Supplementary Table S5). In contrast, the negative relationships between leafing intensity and BCA, though strong at the branch level, were weak and non-significant among species (*r*^2^ < 0.05), indicating that they operate primarily within individuals along the branching hierarchy rather than across species. The positive TLB–branch biomass and BCA–leaf biomass relationships retained the same direction at the species level but, with only 12 species, could not be statistically distinguished from isometry (Supplementary Table S5).

### Plant trait networks across branch orders

**Figure 8:**
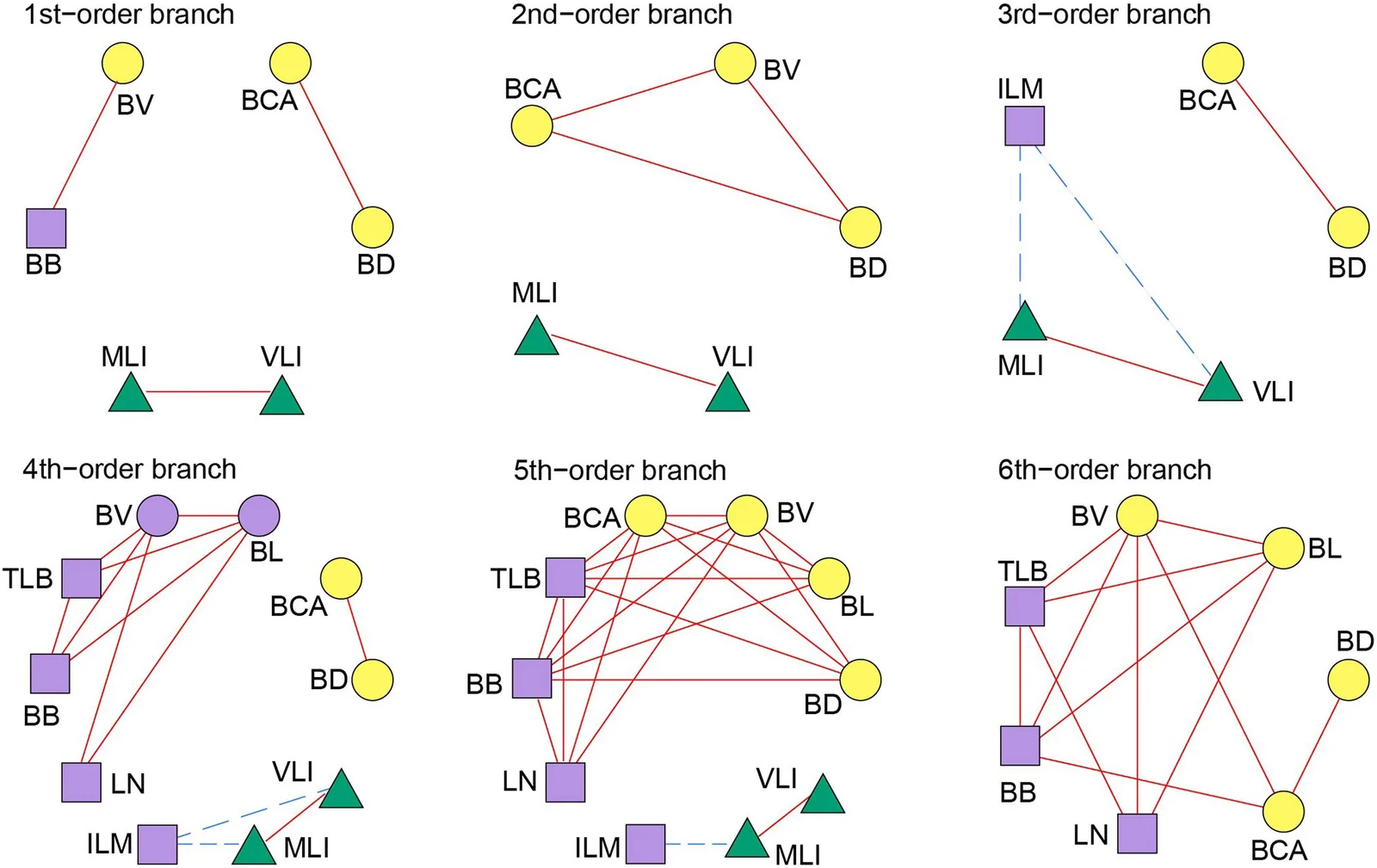
Plant trait networks of branch–leaf traits at successive branch orders (first to sixth order). Nodes are branch–leaf traits and edges are significant Pearson correlations retained at |*r*| > 0.30 and *P* < 0.05. Solid edges denote positive correlations and dashed edges denote negative correlations. Node shape indicates trait category: circles, branch structural traits (BD, branch diameter; BCA, branch cross-sectional area; BL, branch length; BV, branch volume); squares, biomass and leaf-number traits (TLB, total leaf biomass; BB, branch biomass; LN, leaf number; ILM, individual leaf mass); triangles, leafing-intensity traits (MLI, mass-based leafing intensity; VLI, volume-based leafing intensity). Networks become more connected and incorporate more leaf and biomass traits toward higher branch orders.

Plant trait network analysis showed branch-order-dependent changes in the coordination among branch architecture, leaf deployment and biomass allocation (Fig. 8). Lower-order networks were relatively simple: the first-order network mainly comprised BD, BV, BCA, branch biomass, and MLI and VLI and the second-order network mainly comprised BD, BCA, BV, and MLI and VLI. At the third order, ILM and leafing intensity became more prominent, and the fourth- and fifth-order networks incorporated more traits, including BL, BV, TLB, branch biomass, LN, ILM, and MLI and VLI. The sixth-order network was dominated by branch size and biomass traits (BD, BL, BV, BCA, TLB, branch biomass, and LN). Additional networks constructed for architectural life forms, elevational zones, and slope aspects varied in network properties and core traits but were treated as supplementary, as the main objective was to evaluate branch-order-dependent coordination (Supplementary Table S6).

## Discussion

This study aimed to clarify how branching hierarchy structures branch–leaf allometry and trait coordination in shrubs. By combining allometric analysis, phylogenetic diagnostics and plant trait networks across 12 co-occurring shrub species, we show that branch order is not merely a sampling category but a functional level at which structural support, biomass allocation and leaf deployment are coordinated. This perspective is important for botany and plant sciences because shrubs, despite their ecological importance, are often treated as collections of terminal twigs or simplified woody units. Our results suggest that incorporating branching hierarchy can improve comparative studies of plant architecture, allocation, and functional adaptation.

### Phylogenetic background and scale dependence of branch–leaf allometry

Because closely related species often share developmental programmes and allocation strategies, any phylogenetic imprint on traits must be separated from ecological and architectural effects (Harvey and Pagel 1991, Cornwallis and Griffin 2024). In our assemblage, this imprint was weak: most branch–leaf traits showed low or non-significant phylogenetic signal, and species from different life forms, elevational zones, and slope aspects overlapped broadly in phylogenetically corrected trait space. The single exception—a significant Blomberg's *K* for TLB unsupported by Pagel's *λ*—is best treated as equivocal, especially since 12 species give limited power to detect signal. These results do not prove phylogeny irrelevant, but they indicate that relatedness is not the dominant axis structuring branch–leaf variation here, which licences interpreting the following patterns mainly in terms of architecture and environment.

The most consistent feature of the allometry was its dependence on organizational scale. TLB increased disproportionately with branch biomass among whole individuals; yet, the same relationship converged on isometry within individual branches. This is more than a change in slope: it implies that the exponent describing whole-shrub allocation is not a fixed species property but an emergent outcome of summing proportional module-level relationships across a structurally heterogeneous plant. Models that derive universal scaling from an idealized, self-similar branching network (West et al. 1999) assume a near-invariant repeating unit; our results suggest real shrub architectures depart from this, because the balance between supporting and photosynthetic tissue is set locally and only then aggregated. Functionally, a whole shrub must reconcile mechanical stability, water transport, crown expansion, resprouting, and leaf display, and the mechanical and hydraulic costs of spanning greater distances escalate faster than leaf area as a plant enlarges (Tyree and Ewers 1991), biasing whole-plant allocation towards woody tissue under mountain stress. An individual branch carries little of this integrative overhead—its task is mainly to support and supply its own leaves—so a near-proportional coupling of branch and leaf biomass suffices locally. Whole-plant allometry is thus the signature of integration costs that individual modules are largely spared.

BCA reinforced this interpretation. Its positive association with leaf biomass fits its dual role in mechanical support and hydraulic supply (Tyree and Ewers 1991, Smith and Sperry 2014), but the relationship was not governed by a single exponent: slopes ranged from isometric to allometric across ecological groups and branch orders, and both MLI and VLI fell as BCA rose. Thicker, lower-order branches therefore support proportionally fewer leaves and are specialized for transport and spatial deployment, whereas thinner, higher-order branches are specialized for display—a division of labour that also separates the life forms, with densely clustered (Type C) shrubs decentralizing leaf support across many short units and more discrete crowns concentrating it in fewer, larger branches. That one structural variable maps so differently onto leaf support across contexts underscores that shrub branch–leaf allometry is a locally tuned relationship, organized above all by position in the branching hierarchy.

### Leaf size–number trade-off in shrubs

The negative relationships between ILM and both measures of leafing intensity reveal a leaf size–number trade-off throughout these shrubs: leaf area is augmented mainly by adding leaves rather than enlarging them. This is the pattern predicted by the leafing-intensity premium hypothesis, under which selection for many meristems favours smaller leaves (Kleiman and Aarssen 2007), and it matches relationships documented in herbaceous angiosperms, diverse woody floras and temperate trees (Yang et al. 2008, Milla 2009, Scott and Aarssen 2012, Sun et al. 2019). Its recurrence in mountain shrubs suggests a general axis of leaf deployment rather than a growth-form-specific feature, with shrubs sitting towards the many-small-leaves end.

Several features of the shrub life form make this end advantageous. Being short, densely ramified, and disproportionately exposed to near-ground wind, browsing, fire, and abrasion, shrubs build canopies from many inexpensive leaves that buffer piecemeal damage and are cheap to replace (Bellingham and Sparrow 2000, Xie et al. 2019); small leaves also pack efficiently into compact crowns with less self-shading and carry thin boundary layers that dissipate heat and moderate transpiration in open, high-radiation settings (Givnish 1987, Wright et al. 2017). The trade-off also shifted with habitat: subalpine shrubs combined smaller leaves with higher leafing intensity, as expected where low temperature, wind, radiation, and hydraulic limitation favour small leaves whose thin boundary layers keep leaf temperature near air temperature and limit water loss (Parkhurst and Loucks 1972, Givnish and Vermeij 1976), while densely clustered (Type C) shrubs reached high TLB through many small leaves suited to their compact crowns.

Most revealing is that the trade-off reappeared within individuals in asymmetric form. ILM did not vary significantly among branch orders, whereas leafing intensity rose towards higher orders—so redeployment of leaf area along the hierarchy was accomplished almost entirely by varying LN, with leaf size held nearly constant. This implies a developmental division of labour: leaf size behaves as a relatively canalized, species-level property, while LN is the flexible lever through which the plant tunes display to each branch's local role. Because higher-order branches occupy the illuminated canopy periphery, concentrating leaves there raises light capture without the greater construction and hydraulic cost of larger leaves. The trade-off thus operates through a single lever—LN—applied consistently across species, environmental gradients and positions within the plant, tying it directly to the hierarchical control of allocation developed above. Notably, the leaf size–number trade-off held, and was in fact stronger, when species rather than individuals were used as the unit of analysis (Supplementary Table S5), confirming that it is a species-level feature of shrub leaf deployment and not an artefact of pooling autocorrelated individuals.

### Branching hierarchy reshapes trait coordination networks

Pairwise allometries isolate individual relationships but cannot show how the full set of traits is coordinated at once, or which traits hold that coordination together; plant trait networks make both visible (He et al. 2020, Wang et al. 2023). Analysed by branch order, the networks reorganized progressively along the hierarchy. Lower-order networks were sparse and built around branch structural traits and leafing intensity, the signature of units dedicated to support, conduction, and crown extension. Higher-order networks recruited leaf and biomass traits—TLB, branch biomass, LN, ILM, and both leafing-intensity measures—into a more densely connected system in which architecture, leaf display, and biomass allocation were jointly regulated.

That the structure of coordination itself—not merely particular slopes—changes with branch order implies that shrub branches are not equivalent, interchangeable units but functionally differentiated modules whose integration depends on their position in the plant. This accords with the architectural view, in which whole-organism form emerges from the spatial arrangement of modules that change in function as they are produced and age (Barthélémy and Caraglio 2007, Laurans et al. 2024), and it cautions against treating a shrub as a homogeneous population of twigs when scaling from organ to individual. Read together, the three lines of evidence converge from different directions: scale-dependent allometry, a leaf-number-driven size–number trade-off, and order-dependent trait networks all identify branch order as the principal axis along which shrubs balance mechanical and hydraulic support against photosynthetic display. This conclusion should be held with due caution—12 co-occurring species from a single mountain in one season leave phylogenetic and geographic non-independence incompletely excluded, the signal tests are low-powered, and the relationships are correlational—but its consistency across three independent approaches indicates that branching hierarchy is a substantive organizing principle of shrub form, one whose inclusion should sharpen comparative studies of woody-plant allocation, architecture, and adaptation in heterogeneous mountain environments.

## Conclusion

This study shows that branch–leaf allometry and trait coordination in mountain shrubs are organized principally by branching hierarchy. After accounting for phylogeny—which left only a weak imprint on the traits examined—three independent analyses converged: allometry was scale-dependent, shifting from disproportionate scaling of leaf on branch biomass among whole plants to near-proportional scaling within branches; a leaf size–number trade-off ran throughout, with shrubs adjusting display by varying LN while leaf size stayed comparatively fixed; and trait networks reorganized from sparse structural covariation in lower-order branches to integrated coordination in higher-order ones. Together these identify branch order as a key level at which shrubs balance support against display.

Twelve co-occurring species were sufficient to reveal these patterns clearly, and several directions now follow. Extending the approach to more species and independent lineages, and to other biomes and disturbance regimes, would test how general the branch-order signal is and which architectures depart from it. Because the relationships are correlational, directly linking branch order to mechanical and hydraulic function—for example by coupling allometry with measures of xylem transport capacity and safety (Smith and Sperry 2014)—is a natural next step. Finally, the strong intraindividual structuring found here suggests that within-plant variation, rather than being averaged away, merits study in its own right as a window on how modular plants regulate allocation. Explicitly incorporating branching hierarchy should sharpen comparative studies of woody-plant allocation, architecture, and adaptation in heterogeneous environments.

## Supplementary Material

plag041_Supplementary_Data

## Data Availability

The data supporting the findings of this study are openly available in Figshare at https://doi.org/10.6084/m9.figshare.33306168.
