# Supplementary material for: Branching hierarchy shapes branch–leaf allometry and trait coordination in mountain shrubs of Dongling Mountain, Beijing
Source: AoB Plants. 2026 Sep 22;18(5):plag041. doi: 10.1093/aobpla/plag041 (PMC13596049; doi:10.1093/aobpla/plag041)
Supplement: Supplementary file 1 [file plag041_supplementary_data.docx]

**Branching hierarchy shapes branch–leaf allometry and trait coordination in mountain shrubs of Dongling Mountain****, Beijing**

*He Li ^1#^, Siying Zhong ^1#^,* *Xiuchen Jiang ^3^, Jianghua Zhang^2*^*

*^1^* *Jiangsu Normal University, Xuzhou 221000, Jiangsu, China*

*^2^* *XinZhou Normal University, XinZhou 034000, Shanxi, China*

*^3^ Forest Ecosystems and Society, Oregon State University, Corvallis, OR 97331*

ORCID

*He Li: https://orcid.org/0000-0001-9298-6062*

*Siying Zhong: https://orcid.org/0009-0006-4400-6502*

*Xiuchen Jiang: https://orcid.org/0009-0001-9712-9382*

*Jianghua Zhang: https://orcid.org/0000-0001-6338-4759*

^#^These authors contributed equally

^*^ Correspondence:

Jianghua Zhang

Email: wj123-2007@163.com

Tel: 15210676970

**Supplementary Material**

**Table S1.** Phylogenetic signal of branch–leaf traits (Phylogenetic signal was tested using Pagel’s λ and Blomberg’s K based on log10-transformed species-level trait means.)

| **Trait** | **Pagel's λ** | **Pλ** | **Sig.λ** | **Blomberg's K** | **PK** | **Sig.K** |
| --- | --- | --- | --- | --- | --- | --- |
| BD | 0.000 | 1.000 | *ns* | 0.768 | 0.701 | *ns* |
| BCA | 0.000 | 1.000 | *ns* | 0.636 | 0.961 | *ns* |
| BL | 0.000 | 1.000 | *ns* | 0.819 | 0.587 | *ns* |
| BV | 0.000 | 1.000 | *ns* | 0.642 | 0.947 | *ns* |
| SD | 0.000 | 1.000 | *ns* | 0.788 | 0.705 | *ns* |
| TLB | 1.085 | 0.059 | *ns* | 1.297 | 0.009 | * |
| BB | 0.137 | 0.924 | *ns* | 0.938 | 0.293 | *ns* |
| LN | 0.000 | 1.000 | *ns* | 0.898 | 0.342 | *ns* |
| ILM | 0.704 | 0.584 | *ns* | 0.979 | 0.227 | *ns* |
| MLI | 0.000 | 1.000 | *ns* | 0.903 | 0.379 | *ns* |
| VLI | 1.365 | 0.229 | *ns* | 1.068 | 0.098 | *ns* |

Notes: * P < 0.05, Significant phylogenetic signal; *ns*, No significant phylogenetic signal. Trait abbreviations: BD, branch diameter; BCA, branch cross-sectional area; BL, branch length; BV, branch volume; SD, stem density; TLB, total leaf biomass; BB, branch biomass; LN, leaf number; ILM, individual leaf mass; MLI, mass-based leafing intensity; VLI, volume-based leafing intensity.

**Table S2.** Branch and leaf traits across different life forms, elevation zones and slope aspects. Values are presented as mean ± standard error. Different lowercase letters indicate significant differences (*P* < 0.05) among groups for a given trait.

|  | **Life Form** | | | **Elevation Zone** | | | **Aspect** | |
| --- | --- | --- | --- | --- | --- | --- | --- | --- |
|  | Type A | Type B | Type C | Low-mountain zone | Mid-mountain zone | Subalpine zone | Shady | Sunny |
| **BD (cm)** | 0.261 ± 0.029 b | 0.217 ± 0.013 c | 0.337 ± 0.030 a | 0.215 ± 0.011 b | 0.232 ± 0.016 b | 0.439 ± 0.072 a | 0.218 ± 0.012 b | 0.276 ± 0.021 a |
| **BCA (cm²)** | 0.125 ± 0.065 a | 0.055 ± 0.008 c | 0.121 ± 0.022 b | 0.050 ± 0.006 b | 0.060 ± 0.010 b | 0.314 ± 0.167 a | 0.052 ± 0.008 b | 0.118 ± 0.041 a |
| **BL (cm)** | 10.823 ± 1.244 b | 12.853 ± 1.008 a | 14.575 ± 1.427 a | 13.148 ± 0.999 a | 13.343 ± 1.307 a | 7.252 ± 0.799 b | 13.747 ± 1.073 a | 11.415 ± 0.921 a |
| **BV (cm³)** | 1.974 ± 0.729 a | 1.576 ± 0.383 a | 1.326 ± 0.318 b | 1.357 ± 0.407 bc | 1.810 ± 0.482 ac | 2.603 ± 1.320 a | 1.471 ± 0.379 a | 1.830 ± 0.488 a |
| **SD (g cm⁻³)** | 0.645 ± 0.034 b | 0.736 ± 0.031 a | 0.496 ± 0.036 c | 0.631 ± 0.025 ab | 0.737 ± 0.045 a | 0.632 ± 0.038 b | 0.619 ± 0.031 a | 0.700 ± 0.028 a |
| **TLB (g)** | 0.202 ± 0.020 b | 0.212 ± 0.026 b | 0.409 ± 0.040 a | 0.278 ± 0.028 a | 0.186 ± 0.015 a | 0.223 ± 0.034 a | 0.228 ± 0.020 a | 0.250 ± 0.024 a |
| **BB (g)** | 0.751 ± 0.239 a | 0.915 ± 0.212 a | 0.766 ± 0.250 b | 0.723 ± 0.198 a | 1.016 ± 0.250 a | 0.818 ± 0.282 a | 0.776 ± 0.197 a | 0.873 ± 0.193 a |
| **LN** | 1.667 ± 0.128 c | 2.327 ± 0.206 b | 9.128 ± 0.848 a | 3.392 ± 0.380 b | 2.254 ± 0.218 b | 5.152 ± 0.872 a | 3.990 ± 0.427 a | 2.726 ± 0.303 b |
| **ILM (g)** | 0.159 ± 0.017 a | 0.095 ± 0.005 b | 0.057 ± 0.011 c | 0.120 ± 0.010 a | 0.114 ± 0.012 a | 0.061 ± 0.013 b | 0.082 ± 0.009 b | 0.132 ± 0.010 a |
| **MLI (leaves g⁻¹)** | 24.100 ± 2.611 b | 29.048 ± 2.619 b | 44.119 ± 7.608 a | 29.083 ± 2.972 a | 32.194 ± 3.809 a | 28.721 ± 4.084 a | 41.919 ± 3.933 a | 20.416 ± 1.570 b |
| **VLI (leaves cm⁻³)** | 14.651 ± 2.019 b | 20.726 ± 2.208 a | 16.430 ± 1.678 a | 14.390 ± 1.117 a | 23.055 ± 3.180 a | 19.869 ± 3.823 a | 23.132 ± 2.335 a | 13.760 ± 1.359 b |

|  | **Life Form** | | | **Elevation Zone** | | | **Aspect** | |
| --- | --- | --- | --- | --- | --- | --- | --- | --- |
|  | Type A | Type B | Type C | Low-mountain zone | Mid-mountain zone | Subalpine zone | Shady | Sunny |
| **BD (cm)** | 0.261 ± 0.029 b | 0.217 ± 0.013 c | 0.337 ± 0.030 a | 0.215 ± 0.011 b | 0.232 ± 0.016 b | 0.439 ± 0.072 a | 0.218 ± 0.012 b | 0.276 ± 0.021 a |
| **BCA (cm²)** | 0.125 ± 0.065 a | 0.055 ± 0.008 c | 0.121 ± 0.022 b | 0.050 ± 0.006 b | 0.060 ± 0.010 b | 0.314 ± 0.167 a | 0.052 ± 0.008 b | 0.118 ± 0.041 a |
| **BL (cm)** | 10.823 ± 1.244 b | 12.853 ± 1.008 a | 14.575 ± 1.427 a | 13.148 ± 0.999 a | 13.343 ± 1.307 a | 7.252 ± 0.799 b | 13.747 ± 1.073 a | 11.415 ± 0.921 a |
| **BV (cm³)** | 1.974 ± 0.729 a | 1.576 ± 0.383 a | 1.326 ± 0.318 b | 1.357 ± 0.407 bc | 1.810 ± 0.482 ac | 2.603 ± 1.320 a | 1.471 ± 0.379 a | 1.830 ± 0.488 a |
| **SD (g cm⁻³)** | 0.645 ± 0.034 b | 0.736 ± 0.031 a | 0.496 ± 0.036 c | 0.631 ± 0.025 ab | 0.737 ± 0.045 a | 0.632 ± 0.038 b | 0.619 ± 0.031 a | 0.700 ± 0.028 a |
| **TLB (g)** | 0.202 ± 0.020 b | 0.212 ± 0.026 b | 0.409 ± 0.040 a | 0.278 ± 0.028 a | 0.186 ± 0.015 a | 0.223 ± 0.034 a | 0.228 ± 0.020 a | 0.250 ± 0.024 a |
| **BB (g)** | 0.751 ± 0.239 a | 0.915 ± 0.212 a | 0.766 ± 0.250 b | 0.723 ± 0.198 a | 1.016 ± 0.250 a | 0.818 ± 0.282 a | 0.776 ± 0.197 a | 0.873 ± 0.193 a |
| **LN** | 1.667 ± 0.128 c | 2.327 ± 0.206 b | 9.128 ± 0.848 a | 3.392 ± 0.380 b | 2.254 ± 0.218 b | 5.152 ± 0.872 a | 3.990 ± 0.427 a | 2.726 ± 0.303 b |
| **ILM (g)** | 0.159 ± 0.017 a | 0.095 ± 0.005 b | 0.057 ± 0.011 c | 0.120 ± 0.010 a | 0.114 ± 0.012 a | 0.061 ± 0.013 b | 0.082 ± 0.009 b | 0.132 ± 0.010 a |
| **MLI (leaves g⁻¹)** | 24.100 ± 2.611 b | 29.048 ± 2.619 b | 44.119 ± 7.608 a | 29.083 ± 2.972 a | 32.194 ± 3.809 a | 28.721 ± 4.084 a | 41.919 ± 3.933 a | 20.416 ± 1.570 b |
| **VLI (leaves cm⁻³)** | 14.651 ± 2.019 b | 20.726 ± 2.208 a | 16.430 ± 1.678 a | 14.390 ± 1.117 a | 23.055 ± 3.180 a | 19.869 ± 3.823 a | 23.132 ± 2.335 a | 13.760 ± 1.359 b |

Note: BD, branch diameter, BCA, branch cross-sectional area, BL, branch length, BV, branch volume, SD, stem density, TLB, total leaf biomass, BB, branch biomass, LN, leaf number, ILM, individual leaf mass, MLI, mass-based leafing intensity, VLI, volume-based leafing intensity

**Table S3.** Branch and leaf traits across branch orders. Values are presented as mean ± standard error. Different lowercase letters indicate significant differences (*P* < 0.05) among groups for a given trait.

| **Branch Order** | | **1st−order** | **2nd-order** | **3rd-oeder** | **4th-order** | **5th-order** | **6th-order** | **7th-order** |
| --- | --- | --- | --- | --- | --- | --- | --- | --- |
| **BD (cm)** | 0.402 ± 0.023 a | | 0.280 ± 0.040 b | 0.197 ± 0.015 c | 0.152 ± 0.010 d | 0.127 ± 0.008 d | 0.133 ± 0.009 d | 0.124 ± 0.012 d |
| **BCA (cm²)** | 0.158 ± 0.018 a | | 0.150 ± 0.094 b | 0.041 ± 0.009 c | 0.021 ± 0.004 d | 0.014 ± 0.002 d | 0.015 ± 0.002 d | 0.013 ± 0.002 d |
| **BL (cm)** | 22.202 ± 1.979 a | | 11.547 ± 0.998 b | 8.816 ± 0.831 bdef | 7.557 ± 0.959 cdg | 6.655 ± 1.053 ceh | 6.391 ± 1.872 cfi | 7.934 ± 3.076 bghi |
| **BV (cm³)** | 4.776 ± 0.985 a | | 1.479 ± 0.731 b | 0.334 ± 0.074 c | 0.172 ± 0.032 d | 0.125 ± 0.044 d | 0.125 ± 0.055 d | 0.139 ± 0.072 d |
| **SD (g cm⁻³)** | 0.649 ± 0.042 a | | 0.652 ± 0.044 a | 0.629 ± 0.035 a | 0.682 ± 0.045 a | 0.738 ± 0.049 a | 0.584 ± 0.066 a | 1.019 ± 0.394 a |
| **TLB (g)** | 0.423 ± 0.065 a | | 0.232 ± 0.027 b | 0.211 ± 0.025 b | 0.184 ± 0.028 b | 0.175 ± 0.042 b | 0.141 ± 0.036 b | 0.175 ± 0.063 b |
| **BB (g)** | 2.683 ± 0.489 a | | 0.424 ± 0.075 b | 0.147 ± 0.022 c | 0.091 ± 0.015 d | 0.062 ± 0.013 d | 0.051 ± 0.017 d | 0.065 ± 0.022 cd |
| **LN** | 5.513 ± 0.976 a | | 3.211 ± 0.428 bc | 2.745 ± 0.367 be | 2.114 ± 0.292 bg | 2.875 ± 0.854 bd | 2.556 ± 0.973 bf | 3.500 ± 1.432 acdefg |
| **ILM (g)** | 0.146 ± 0.024 a | | 0.125 ± 0.016 ac | 0.099 ± 0.011 ad | 0.093 ± 0.010 ae | 0.090 ± 0.015 af | 0.061 ± 0.007 bcdefg | 0.065 ± 0.011 ag |
| **MLI (leaves g⁻¹)** | 10.298 ± 1.646 d | | 26.009 ± 5.620 c | 29.393 ± 3.081 be | 35.242 ± 3.478 ae | 50.974 ± 7.690 a | 50.066 ± 6.288 a | 57.762 ± 7.151 a |
| **VLI (leaves cm⁻³)** | 4.962 ± 0.606 d | | 12.657 ± 1.835 c | 16.777 ± 1.986 bef | 23.158 ± 2.995 af | 37.427 ± 7.295 a | 28.984 ± 5.597 ae | 35.604 ± 8.148 a |

Note: Traits of 8th–9th order branches were not compared due to insufficient sample size (*n* < 5), resulting in large standard errors. BD, branch diameter, BCA, branch cross-sectional area, BL, branch length, BV, branch volume, SD, stem density, TLB, total leaf biomass, BB, branch biomass, LN, leaf number, ILM, individual leaf mass, MLI, mass-based leafing intensity, VLI, volume-based leafing intensity

**Table S4. Standardized major axis regression results for branch–leaf allometric relationships.**

| **Relationship** | **Analysis level** | **Grouping factor** | **Group** | **Predictor (X)** | **Response (Y)** | **SMA slope (α)** | **Lower 95% CI** | **Upper 95% CI** | **Intercept** | **r** | **R²** | ***P* value** | **Expected slope** | **Scaling type** |
| --- | --- | --- | --- | --- | --- | --- | --- | --- | --- | --- | --- | --- | --- | --- |
| Individual level: leaf biomass vs branch biomass | Individual level | Overall | All individuals | BB | TLB | 0.536 | 0.436 | 0.659 | -0.27 | 0.462 | 0.213 | <0.001* | 1 | Negative allometry |
| Individual level: leaf biomass vs branch biomass | Individual level | Elevation zone | Low-mountain zone | BB | TLB | 0.64 | 0.482 | 0.851 | -0.19 | 0.545 | 0.297 | <0.001* | 1 | Negative allometry |
| Individual level: leaf biomass vs branch biomass | Individual level | Elevation zone | Mid-mountain zone | BB | TLB | 0.316 | 0.214 | 0.468 | -0.33 | 0.368 | 0.135 | 0.071ns | 1 | Negative allometry |
| Individual level: leaf biomass vs branch biomass | Individual level | Elevation zone | Subalpine zone | BB | TLB | 0.588 | 0.361 | 0.957 | -0.36 | 0.694 | 0.482 | 0.012* | 1 | Negative allometry |
| Individual level: leaf biomass vs branch biomass | Individual level | Life form | Type A | BB | TLB | 0.472 | 0.337 | 0.661 | -0.3 | 0.464 | 0.215 | 0.01* | 1 | Negative allometry |
| Individual level: leaf biomass vs branch biomass | Individual level | Life form | Type B | BB | TLB | 0.515 | 0.373 | 0.712 | -0.33 | 0.467 | 0.218 | 0.007* | 1 | Negative allometry |
| Individual level: leaf biomass vs branch biomass | Individual level | Life form | Type C | BB | TLB | 0.552 | 0.3 | 1.016 | -0.05 | 0.389 | 0.151 | 0.212ns | 1 | Isometric |
| Individual level: leaf biomass vs branch biomass | Individual level | Slope aspect | Sunny slope | BB | TLB | 0.548 | 0.424 | 0.71 | -0.27 | 0.563 | 0.317 | <0.001* | 1 | Negative allometry |
| Individual level: leaf biomass vs branch biomass | Individual level | Slope aspect | Shady slope | BB | TLB | 0.517 | 0.364 | 0.735 | -0.29 | 0.336 | 0.113 | 0.065ns | 1 | Negative allometry |
| Individual level: leaf biomass vs branch biomass | Branch level | Branch position | Primary branch | BB | TLB | 0.81 | 0.614 | 1.069 | -0.35 | 0.43 | 0.185 | 0.004* | 1 | Isometric |
| Individual level: leaf biomass vs branch biomass | Branch level | Branch position | Terminal branch | BB | TLB | 0.808 | 0.692 | 0.943 | 0.244 | 0.754 | 0.569 | <0.001* | 1 | Negative allometry |
| Branch level: total leaf biomass vs branch cross-sectional area | Branch level | Overall | All species/branches | BCA | TLB | 0.868 | 0.776 | 0.971 | 0.571 | 0.416 | 0.173 | <0.001* | 1 | Negative allometry |
| Branch level: total leaf biomass vs branch cross-sectional area | Branch level | Elevation zone | Low-mountain zone | BCA | TLB | 1.318 | 1.134 | 1.531 | 1.395 | 0.458 | 0.21 | <0.001* | 1 | Positive allometry |
| Branch level: total leaf biomass vs branch cross-sectional area | Branch level | Elevation zone | Mid-mountain zone | BCA | TLB | 0.822 | 0.682 | 0.991 | 0.542 | 0.545 | 0.297 | <0.001* | 1 | Negative allometry |
| Branch level: total leaf biomass vs branch cross-sectional area | Branch level | Elevation zone | Subalpine zone | BCA | TLB | 0.461 | 0.343 | 0.62 | -0.26 | 0.483 | 0.234 | 0.002* | 1 | Negative allometry |
| Branch level: total leaf biomass vs branch cross-sectional area | Branch level | Life form | Type A | BCA | TLB | 0.817 | 0.672 | 0.993 | 0.442 | 0.352 | 0.124 | <0.001* | 1 | Negative allometry |
| Branch level: total leaf biomass vs branch cross-sectional area | Branch level | Life form | Type B | BCA | TLB | 1.082 | 0.914 | 1.28 | 0.976 | 0.355 | 0.126 | <0.001* | 1 | Isometric |
| Branch level: total leaf biomass vs branch cross-sectional area | Branch level | Life form | Type C | BCA | TLB | 0.551 | 0.405 | 0.75 | 0.194 | 0.197 | 0.039 | 0.211ns | 1 | Negative allometry |
| Branch level: total leaf biomass vs branch cross-sectional area | Branch level | Slope aspect | Sunny slope | BCA | TLB | 0.792 | 0.683 | 0.919 | 0.395 | 0.382 | 0.146 | <0.001* | 1 | Negative allometry |
| Branch level: total leaf biomass vs branch cross-sectional area | Branch level | Slope aspect | Shady slope | BCA | TLB | 1.181 | 1.004 | 1.388 | 1.187 | 0.561 | 0.315 | <0.001* | 1 | Positive allometry |
| Branch level: total leaf biomass vs branch cross-sectional area | Branch level | Branch position | Primary branch | BCA | TLB | 1.112 | 0.837 | 1.476 | 0.732 | 0.384 | 0.147 | 0.01* | 1 | Isometric |
| Branch level: total leaf biomass vs branch cross-sectional area | Branch level | Branch position | Terminal branch | BCA | TLB | 0.837 | 0.693 | 1.012 | 0.666 | 0.593 | 0.351 | <0.001* | 1 | Isometric |
| Branch level: mass-based leafing intensity vs branch cross-sectional area | Branch level | Overall | All species/branches | BCA | MLI | -1.12 | -1.25 | -1.01 | -0.51 | -0.59 | 0.348 | <0.001* | -1 | More negative than expected |
| Branch level: mass-based leafing intensity vs branch cross-sectional area | Branch level | Elevation zone | Low-mountain zone | BCA | MLI | -1.52 | -1.74 | -1.33 | -1.24 | -0.66 | 0.432 | <0.001* | -1 | More negative than expected |
| Branch level: mass-based leafing intensity vs branch cross-sectional area | Branch level | Elevation zone | Mid-mountain zone | BCA | MLI | -1.47 | -1.68 | -1.29 | -1.22 | -0.84 | 0.703 | <0.001* | -1 | More negative than expected |
| Branch level: mass-based leafing intensity vs branch cross-sectional area | Branch level | Elevation zone | Subalpine zone | BCA | MLI | -0.69 | -0.93 | -0.52 | 0.508 | -0.59 | 0.348 | <0.001* | -1 | Less negative than expected |
| Branch level: mass-based leafing intensity vs branch cross-sectional area | Branch level | Life form | Type A | BCA | MLI | -1.24 | -1.49 | -1.03 | -0.75 | -0.59 | 0.349 | <0.001* | -1 | More negative than expected |
| Branch level: mass-based leafing intensity vs branch cross-sectional area | Branch level | Life form | Type B | BCA | MLI | -1.32 | -1.48 | -1.17 | -1.01 | -0.79 | 0.616 | <0.001* | -1 | More negative than expected |
| Branch level: mass-based leafing intensity vs branch cross-sectional area | Branch level | Life form | Type C | BCA | MLI | -0.92 | -1.13 | -0.75 | 0.308 | -0.78 | 0.606 | <0.001* | -1 | Isometric |
| Branch level: mass-based leafing intensity vs branch cross-sectional area | Branch level | Slope aspect | Sunny slope | BCA | MLI | -0.94 | -1.09 | -0.81 | -0.28 | -0.53 | 0.279 | <0.001* | -1 | Isometric |
| Branch level: mass-based leafing intensity vs branch cross-sectional area | Branch level | Slope aspect | Shady slope | BCA | MLI | -1.46 | -1.69 | -1.26 | -1.02 | -0.67 | 0.453 | <0.001* | -1 | More negative than expected |
| Branch level: mass-based leafing intensity vs branch cross-sectional area | Branch level | Branch position | Primary branch | BCA | MLI | -1.32 | -1.79 | -0.97 | -0.78 | -0.38 | 0.142 | 0.018* | -1 | Isometric |
| Branch level: mass-based leafing intensity vs branch cross-sectional area | Branch level | Branch position | Terminal branch | BCA | MLI | -0.85 | -1.08 | -0.68 | 0.057 | -0.44 | 0.19 | <0.001* | -1 | Isometric |
| Branch level: volume-based leafing intensity vs branch cross-sectional area | Branch level | Overall | All species/branches | BCA | VLI | -1.2 | -1.31 | -1.1 | -0.89 | -0.76 | 0.577 | <0.001* | -1 | More negative than expected |
| Branch level: volume-based leafing intensity vs branch cross-sectional area | Branch level | Elevation zone | Low-mountain zone | BCA | VLI | -1.42 | -1.57 | -1.28 | -1.34 | -0.84 | 0.702 | <0.001* | -1 | More negative than expected |
| Branch level: volume-based leafing intensity vs branch cross-sectional area | Branch level | Elevation zone | Mid-mountain zone | BCA | VLI | -1.6 | -1.76 | -1.46 | -1.64 | -0.92 | 0.853 | <0.001* | -1 | More negative than expected |
| Branch level: volume-based leafing intensity vs branch cross-sectional area | Branch level | Elevation zone | Subalpine zone | BCA | VLI | -1.02 | -1.28 | -0.82 | -0.16 | -0.78 | 0.609 | <0.001* | -1 | Isometric |
| Branch level: volume-based leafing intensity vs branch cross-sectional area | Branch level | Life form | Type A | BCA | VLI | -1.45 | -1.6 | -1.31 | -1.36 | -0.9 | 0.818 | <0.001* | -1 | More negative than expected |
| Branch level: volume-based leafing intensity vs branch cross-sectional area | Branch level | Life form | Type B | BCA | VLI | -1.43 | -1.56 | -1.32 | -1.39 | -0.9 | 0.808 | <0.001* | -1 | More negative than expected |
| Branch level: volume-based leafing intensity vs branch cross-sectional area | Branch level | Life form | Type C | BCA | VLI | -0.7 | -0.89 | -0.54 | 0.234 | -0.66 | 0.431 | <0.001* | -1 | Less negative than expected |
| Branch level: volume-based leafing intensity vs branch cross-sectional area | Branch level | Slope aspect | Sunny slope | BCA | VLI | -1.11 | -1.26 | -0.97 | -0.77 | -0.7 | 0.496 | <0.001* | -1 | Isometric |
| Branch level: volume-based leafing intensity vs branch cross-sectional area | Branch level | Slope aspect | Shady slope | BCA | VLI | -1.41 | -1.56 | -1.28 | -1.21 | -0.87 | 0.749 | <0.001* | -1 | More negative than expected |
| Branch level: volume-based leafing intensity vs branch cross-sectional area | Branch level | Branch position | Primary branch | BCA | VLI | -1.19 | -1.6 | -0.88 | -0.88 | -0.42 | 0.173 | 0.009* | -1 | Isometric |
| Branch level: volume-based leafing intensity vs branch cross-sectional area | Branch level | Branch position | Terminal branch | BCA | VLI | -0.89 | -1.11 | -0.71 | -0.23 | -0.54 | 0.286 | <0.001* | -1 | Isometric |
| Branch level: individual leaf mass vs mass-based leafing intensity | Branch level | Overall | All species/branches | MLI | ILM | -0.61 | -0.68 | -0.55 | -0.31 | -0.53 | 0.276 | <0.001* | -1 | Less negative than expected |
| Branch level: individual leaf mass vs mass-based leafing intensity | Branch level | Elevation zone | Low-mountain zone | MLI | ILM | -0.62 | -0.72 | -0.54 | -0.25 | -0.57 | 0.327 | <0.001* | -1 | Less negative than expected |
| Branch level: individual leaf mass vs mass-based leafing intensity | Branch level | Elevation zone | Mid-mountain zone | MLI | ILM | -0.56 | -0.68 | -0.46 | -0.36 | -0.53 | 0.276 | <0.001* | -1 | Less negative than expected |
| Branch level: individual leaf mass vs mass-based leafing intensity | Branch level | Elevation zone | Subalpine zone | MLI | ILM | -0.46 | -0.63 | -0.34 | -0.71 | -0.48 | 0.234 | 0.004* | -1 | Less negative than expected |
| Branch level: individual leaf mass vs mass-based leafing intensity | Branch level | Life form | Type A | MLI | ILM | -0.69 | -0.83 | -0.58 | -0.17 | -0.63 | 0.398 | <0.001* | -1 | Less negative than expected |
| Branch level: individual leaf mass vs mass-based leafing intensity | Branch level | Life form | Type B | MLI | ILM | -0.5 | -0.59 | -0.41 | -0.45 | -0.34 | 0.116 | <0.001* | -1 | Less negative than expected |
| Branch level: individual leaf mass vs mass-based leafing intensity | Branch level | Life form | Type C | MLI | ILM | -0.41 | -0.54 | -0.3 | -0.73 | -0.46 | 0.211 | 0.003* | -1 | Less negative than expected |
| Branch level: individual leaf mass vs mass-based leafing intensity | Branch level | Slope aspect | Sunny slope | MLI | ILM | -0.65 | -0.77 | -0.56 | -0.26 | -0.44 | 0.196 | <0.001* | -1 | Less negative than expected |
| Branch level: individual leaf mass vs mass-based leafing intensity | Branch level | Slope aspect | Shady slope | MLI | ILM | -0.54 | -0.64 | -0.46 | -0.42 | -0.52 | 0.269 | <0.001* | -1 | Less negative than expected |
| Branch level: individual leaf mass vs mass-based leafing intensity | Branch level | Branch position | Primary branch | MLI | ILM | -0.75 | -0.99 | -0.56 | -0.42 | -0.51 | 0.26 | <0.001* | -1 | Less negative than expected |
| Branch level: individual leaf mass vs mass-based leafing intensity | Branch level | Branch position | Terminal branch | MLI | ILM | -0.95 | -1.16 | -0.77 | 0.433 | -0.63 | 0.4 | <0.001* | -1 | Isometric |
| Branch level: individual leaf mass vs volume-based leafing intensity | Branch level | Overall | All species/branches | VLI | ILM | -0.57 | -0.64 | -0.51 | -0.51 | -0.46 | 0.214 | <0.001* | -1 | Less negative than expected |
| Branch level: individual leaf mass vs volume-based leafing intensity | Branch level | Elevation zone | Low-mountain zone | VLI | ILM | -0.67 | -0.78 | -0.58 | -0.38 | -0.57 | 0.325 | <0.001* | -1 | Less negative than expected |
| Branch level: individual leaf mass vs volume-based leafing intensity | Branch level | Elevation zone | Mid-mountain zone | VLI | ILM | -0.51 | -0.63 | -0.42 | -0.52 | -0.47 | 0.221 | <0.001* | -1 | Less negative than expected |
| Branch level: individual leaf mass vs volume-based leafing intensity | Branch level | Elevation zone | Subalpine zone | VLI | ILM | -0.31 | -0.44 | -0.22 | -0.99 | -0.37 | 0.138 | 0.034* | -1 | Less negative than expected |
| Branch level: individual leaf mass vs volume-based leafing intensity | Branch level | Life form | Type A | VLI | ILM | -0.59 | -0.72 | -0.48 | -0.45 | -0.45 | 0.199 | <0.001* | -1 | Less negative than expected |
| Branch level: individual leaf mass vs volume-based leafing intensity | Branch level | Life form | Type B | VLI | ILM | -0.46 | -0.54 | -0.38 | -0.59 | -0.42 | 0.174 | <0.001* | -1 | Less negative than expected |
| Branch level: individual leaf mass vs volume-based leafing intensity | Branch level | Life form | Type C | VLI | ILM | -0.53 | -0.69 | -0.41 | -0.73 | -0.61 | 0.377 | <0.001* | -1 | Less negative than expected |
| Branch level: individual leaf mass vs volume-based leafing intensity | Branch level | Slope aspect | Sunny slope | VLI | ILM | -0.56 | -0.65 | -0.47 | -0.5 | -0.42 | 0.178 | <0.001* | -1 | Less negative than expected |
| Branch level: individual leaf mass vs volume-based leafing intensity | Branch level | Slope aspect | Shady slope | VLI | ILM | -0.56 | -0.67 | -0.47 | -0.54 | -0.4 | 0.157 | <0.001* | -1 | Less negative than expected |
| Branch level: individual leaf mass vs volume-based leafing intensity | Branch level | Branch position | Primary branch | VLI | ILM | -0.83 | -1.09 | -0.63 | -0.57 | -0.56 | 0.308 | <0.001* | -1 | Isometric |
| Branch level: individual leaf mass vs volume-based leafing intensity | Branch level | Branch position | Terminal branch | VLI | ILM | -0.91 | -1.12 | -0.74 | 0.173 | -0.62 | 0.381 | <0.001* | -1 | Isometric |

Notes: SMA regressions were fitted using log10-transformed trait values. Regression significance refers to the Pearson correlation underlying the SMA relationship. Significance is coded as * for P < 0.05 and *ns* for P ≥ 0.05.

**Table S5.** Standardized major axis (SMA) regression of branch–leaf allometric relationships fitted at the branch, individual (whole-plant), and species-mean levels. All variables were log_10_-transformed before analysis; species-mean values are geometric means across each species’ records (*n* = 12 species). The species-mean fits test whether the relationships are robust to non-independence among conspecific individuals.

| Relationship (y vs x) | Level | n | Slope | 95% CI | Intercept | *r*^2^ | *P* | H0: slope = 1 |
| --- | --- | --- | --- | --- | --- | --- | --- | --- |
| *Total leaf biomass vs branch biomass* | Branch | 256 | 0.672 | 0.605 to 0.747 | -0.172 | 0.273 | < 0.001 | Allometric (1 outside CI) |
|  | Individual (whole plant) | 74 | 0.536 | 0.436 to 0.659 | -0.274 | 0.213 | < 0.001 | Allometric (1 outside CI) |
|  | Species mean | 12 | 0.770 | 0.415 to 1.431 | -0.310 | 0.123 | 0.263 | Isometric (1 within CI) |
| *Branch cross-sectional area vs total leaf biomass* | Branch | 256 | 0.868 | 0.776 to 0.971 | 0.571 | 0.173 | < 0.001 | Allometric (1 outside CI) |
|  | Species mean | 12 | 0.995 | 0.542 to 1.826 | 0.667 | 0.161 | 0.195 | Isometric (1 within CI) |
| *Mass-based leafing intensity vs branch cross-sectional area* | Branch | 224 | -1.121 | -1.248 to -1.008 | -0.509 | 0.348 | < 0.001 | n.a. |
|  | Species mean | 12 | -1.071 | -2.044 to -0.561 | -0.355 | 0.035 | 0.560 | n.a. |
| *Volume-based leafing intensity vs branch cross-sectional area* | Branch | 224 | -1.203 | -1.311 to -1.104 | -0.889 | 0.577 | < 0.001 | n.a. |
|  | Species mean | 12 | -0.959 | -1.827 to -0.503 | -0.436 | 0.041 | 0.529 | n.a. |
| *Individual leaf mass vs mass-based leafing intensity* | Branch | 224 | -0.610 | -0.682 to -0.545 | -0.314 | 0.276 | < 0.001 | n.a. |
|  | Species mean | 12 | -1.095 | -1.583 to -0.758 | 0.289 | 0.714 | < 0.001 | n.a. |
| *Individual leaf mass vs volume-based leafing intensity* | Branch | 224 | -0.568 | -0.639 to -0.506 | -0.509 | 0.214 | < 0.001 | n.a. |
|  | Species mean | 12 | -1.223 | -1.799 to -0.832 | 0.144 | 0.686 | < 0.001 | n.a. |

Notes: Slope is the SMA (reduced major axis) slope with its 95% confidence interval (CI) sensu Warton et al. (2006). P tests the null hypothesis of no association (H0: correlation = 0; df = n-2).The final column reports the isometry test for the positive size–size relationships (H0: slope = 1): “Allometric” indicates 1.0 lies outside the 95% CI and “Isometric” that it lies within. This test is not applicable (n.a.) to the negative leafing-intensity and leaf-size relationships, for which the relevant contrast is a slope of 0 (see P). At the species level the leaf size–number trade-off (individual leaf mass vs leafing intensity) remained strong and significant, whereas the leafing-intensity–branch cross-sectional area relationships became weak and non-significant, indicating that the latter operate within individuals along the branching hierarchy rather than among species. The positive biomass and area relationships retained their direction but, with only 12 species, could not be statistically distinguished from isometry.

**Table S6.** Network-level metrics and core traits of life-form, elevation zone and Slope aspect- specific PTNs.

| **Groups** |  | **No. of edges** | **Positive edges** | **Negative edges** | **Edge density** | **Diameter** | **Average path length** | **Average clustering coefficient** | **Modularity** | **Core traits** |
| --- | --- | --- | --- | --- | --- | --- | --- | --- | --- | --- |
| **Life form** | Type A | 29 | 17 | 12 | 0.644 | 3.000 | 1.400 | 0.760 | 0.012 | BV, BD, MLI, VLI |
|  | Type B | 37 | 23 | 14 | 0.673 | 3.000 | 1.345 | 0.831 | 0.000 | TLB, VLI, BD, BL |
|  | Type C | 19 | 10 | 9 | 0.422 | 5.000 | 2.000 | 0.739 | 0.159 | BD, BV, MLI, BCA |
| **Elevation zone** | Low-mountain zone | 5 | 5 | 0 | 0.238 | 1.000 | 1.000 | 1.000 | 0.560 | TLB, BB, LN, BV |
|  | mid-mountain zone | 37 | 22 | 15 | 0.822 | 2.000 | 1.178 | 0.845 | 0.000 | MLI, VLI, TLB, BD |
|  | Subalpine zone | 41 | 25 | 16 | 0.745 | 2.000 | 1.255 | 0.783 | 0.019 | BD, BV, MLI, BL |
| **Slope aspect** | Sunny | 38 | 22 | 16 | 0.691 | 3.000 | 1.327 | 0.737 | 0.000 | BD, BV, TLB, BB |
|  | Shady | 32 | 19 | 13 | 0.582 | 3.000 | 1.436 | 0.719 | 0.051 | BD, BV, MLI, VLI |

Note:Network edges represent significant Pearson correlations retained at |*r*| > 0.30 and *P* < 0.05. Core traits were identified as the top four traits ranked by node degree and betweenness centrality. BD, branch diameter, BCA, branch cross-sectional area, BL, branch length, BV, branch volume, SD, stem density, TLB, total leaf biomass, BB, branch biomass, LN, leaf number, ILM, individual leaf mass, MLI, mass-based leafing intensity, VLI, volume-based leafing intensity
